# Insights into Hip pain using Hip X-ray: Epidemiological study of 8,898,044 Koreans

**DOI:** 10.1038/s41598-024-70259-z

**Published:** 2024-08-21

**Authors:** Taewook Kim, Yoonhee Kim, Woosup Cho

**Affiliations:** 1 Department of Orthopedic Surgery, Seoul National University Hospital, Seoul, Korea; 2 Department of Rehabilitation Medicine, Seoul National University Bundang Hospital, Seongnam, Republic of Korea; 3 Department of Rehabilitation Medicine, Armed Forces Medical Command, Armed Forces Yangju Hospital, 460-3, Yongam-ri, Eunhyeon-myeon, Yangju-si, Gyeonggi-do Republic of Korea

**Keywords:** Diseases, Health care, Medical research, Risk factors, Signs and symptoms

## Abstract

Hip pain is a prevalent degenerative joint symptoms, imposing a significant global health burden. Hip pain is experiencing an increase in incidences in Korea due to its aging society, and the social burden of hip pain continues to rise as the hip joint is crucial for gait and balance. This study assessed the epidemiology of hip pain in Korea using data from the fifth version of Korea National Health and Nutrition Examination Survey (KNHANES V-5). The research analyzed data from 8,898,044 Koreans to evaluate the prevalence and characteristics of hip pain and abnormal hip X-ray. Variables encompassed medical, demographic, mental, social, and musculoskeletal factors. Descriptive analysis and propensity score matching analyses unveiled characteristics of Koreans experiencing hip pain or showing abnormal hip x-ray. The study provides insights into the epidemiology of hip pain in the entire Korean population, and further suggesting the effective management of hip pain.

## Introduction

Hip pain is a widespread and disabling issue affecting individuals of all ages. It is mostly characterized by the gradual deterioration of cartilage within the joint and its surrounding structures, which poses significant health challenges. Patients often experience localized pain in three specific areas: the groin, buttock, or the side of the hip. Pain in the groin area is frequently associated with osteoarthritis (OA) and hip labral tears. Pain in the buttock region may be caused by sacroiliac joint dysfunction, lumbar radiculopathy, and less commonly, ischiofemoral impingement. Pain on the lateral side of the hip is typically related to greater trochanteric pain syndrome. The prevalence of hip pain related diseases, such as OA, is estimated to be over 7% of the global population, with cases increasing by 113.25% from 247.51 million in 1990 to 527.81 million in 2019^1,2^.

The hip joint, subjected to the highest torque and force during standing and walking, bears a substantial load, equivalent to approximately 300% of body weight^3^. Considering the role of the hip joint in gait and balance, individuals with hip pain experience limitations in daily activities, leading to a diminished quality of life^4^. Prolonged aging in Korea contributes to cumulative hip joint damage, resulting in a 47% increase in annual incidences of primary total hip arthroplasties from 2010 to 2019^5^.

Despite the introduction of various treatment methods such as medication, surgery, and mesenchymal stem cell injection, the social burden of hip pain continues to rise^6,7^. The increasing social burden of hip pain has brought attention to the need of research for innovative approaches and interventions.

This study investigated data from the fifth version of the Korea National Health and Nutrition Examination Survey (KNHANES V-5), based on a 10,000-sample equivalent to medical insurance claim records from approximately 50 million enrollees, systematically sampled records across the entire Korean population by region, age group, or other factors^8^. Utilizing precise sampling weights, we analyzed data from 8,898,044 individuals with diverse characteristics, including Kellgren-Lawrence grade of hip X-ray, health survey data, socio-economic indicators, and demographic information. By comparing the characteristics between individuals with hip pain and those with hip X-ray, we aim to provide valuable insights for the effective management of hip pain.

## Method

### Data source

Our study enrolled participants from the fifth version of the Korea National Health and Nutrition Examination Survey (KNHANES V-5), a yearly survey conducted between 2010 and 2011. KNHANES is a cross-sectional study started in 1998 to evaluate the health status of the Korean population. Administered by the Korea disease control and prevention agency (KDCA), the survey annually includes approximately 10,000 participants selected from 3,840 households across 192 regions in South Korea. Employing a two-stage sampling approach based on living region and age, involving stratification, clustering, and weighting, KNHANES, when adjusted for sampling weights, is representative of the entire Korean population (N = 46,286,503). The careful application of sampling weights ensures that calculated estimates accurately reflect Korean population characteristics. Further details on the process of obtaining sampling weights were provided in relevant literature^8^.

**Figure 1:**
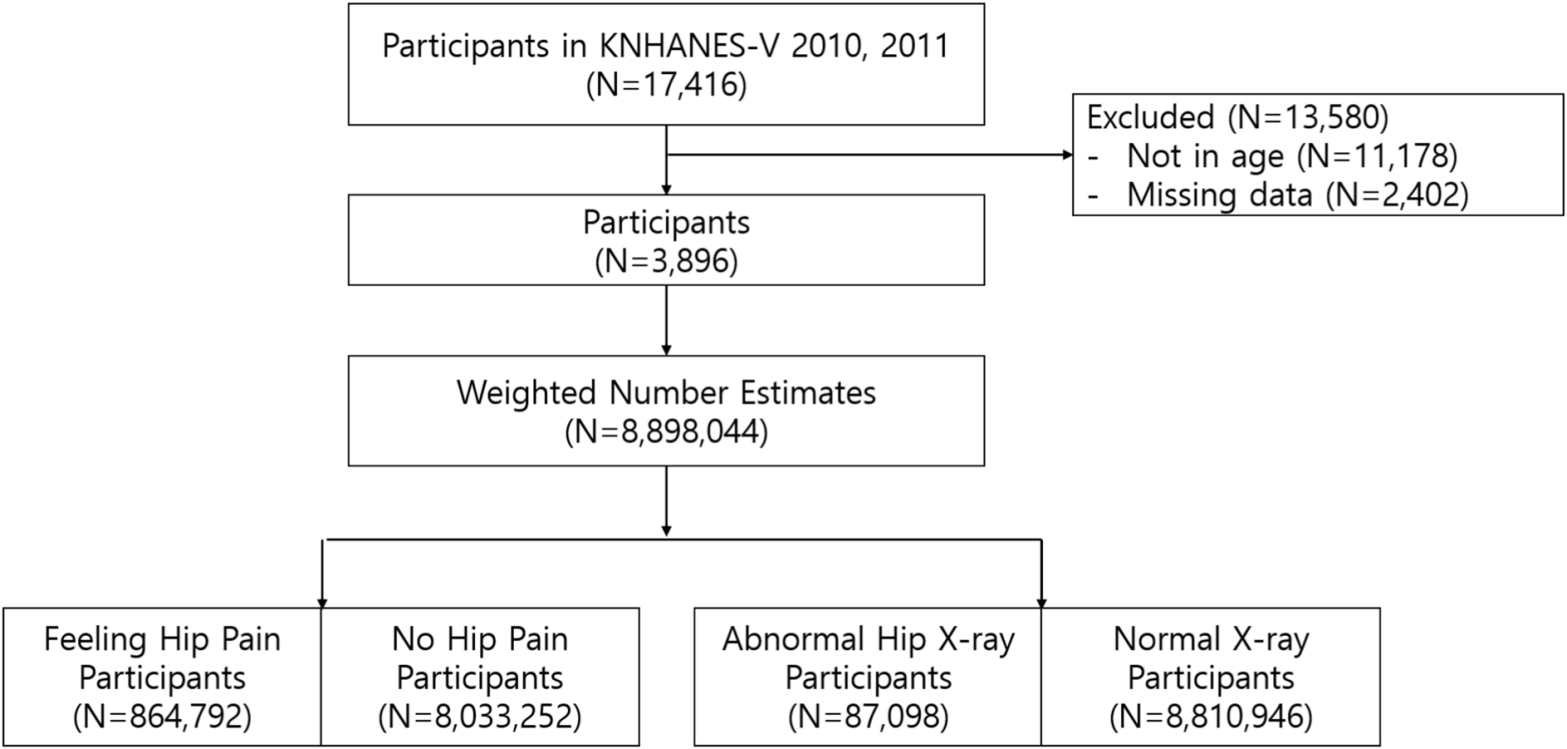
Study design of the study.

Figure 1 illustrates our participant selection process, which began with 17,476 participants from the KNHANES 2010–2011. We excluded participants with missing or inaccurate data, those who did not undergo hip plain radiographs (N = 2,402), and those aged over 80 or below 50 (N = 11,178). Eventually, we analyzed data from 3896 participants aged 50–79. To ensure the representativeness of all Koreans, we utilized complex sampling weights, and the weighted number estimate was assessed in this study (N = 8,898,044). The proper usage of sampling weights ensures that calculated estimates are truly representative of the Korean population. We divided the weighted number estimate into the "Severe grade hip x-ray group" and the normal x-ray group to identify characteristics. "Severe hip x-ray group" was defined as participants of plain radiographs showing Kellgren-Lawrence grade ≥ 2^9^. Furthermore, we divided the weighted number estimate into the "Feeling hip pain group" and the "no hip pain group" to identify characteristics related to hip pain.

### Characteristics

**Figure 2:**
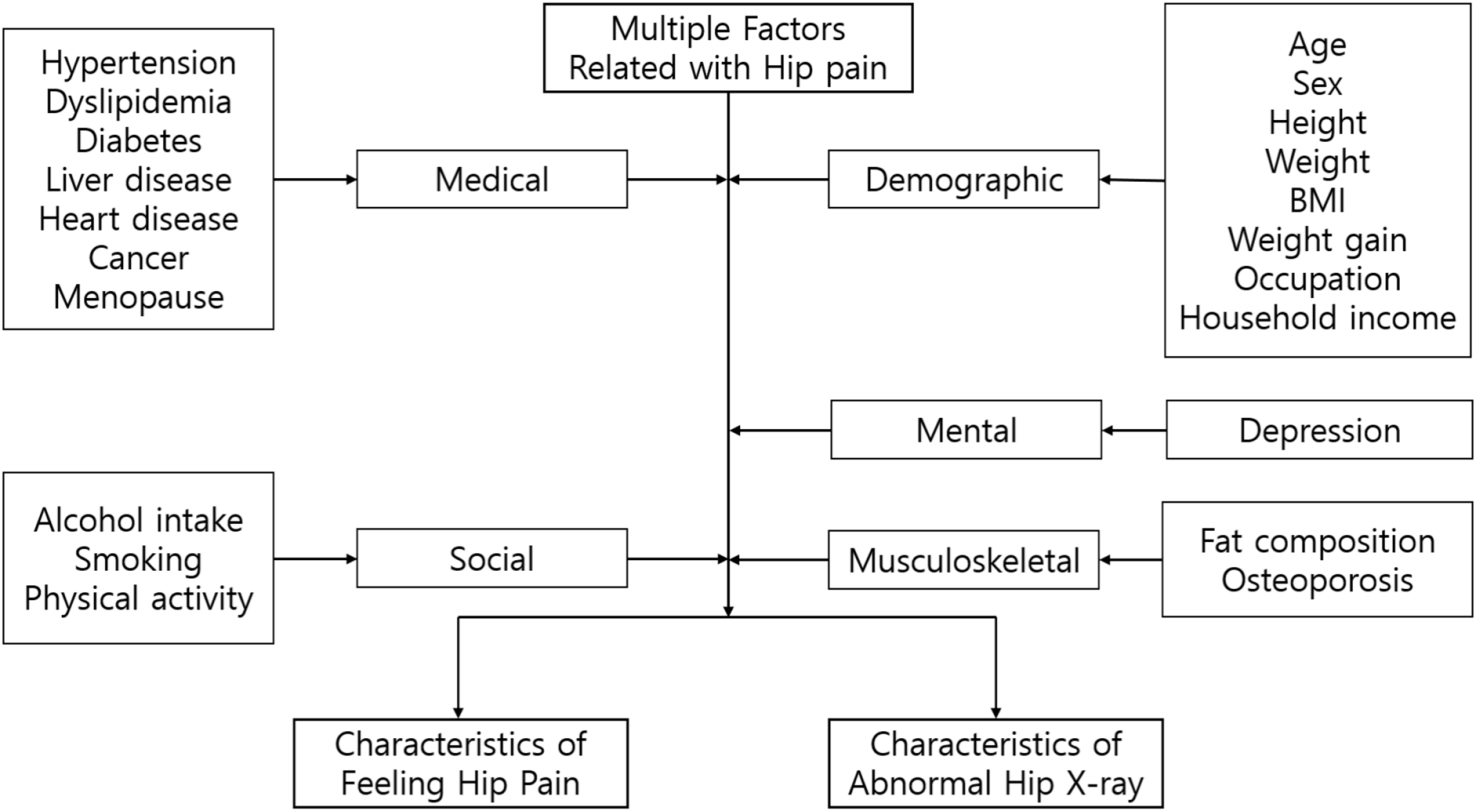
Multiple type of characteristics for epidemiologic analysis of hip pain in Korea.

Figure 2 illustrates the characteristics utilized in this research. Medical and mental factors consist of diagnosed diseases based on health surveys conducted by trained staff members. The KNHANES survey includes the current diagnosis of hypertension, dyslipidemia, diabetes, and cardiovascular disease (heart disease). Additionally, this study incorporates depression as a risk factor, defined by a persistent depressive mood lasting over 2 weeks. The exact questionnaire for assessing depressive mood in this study is: "Have you experienced feeling sad or hopeless to the extent that it significantly interfered with your daily life for two weeks or more continuously in the past year?” Based on previous studies, these medical and mental factors were considered potential risk factors for hip pain^10^.

Musculoskeletal factors, such as fat compositions measured by dual-energy X-ray absorptiometry (DXA), were collected at the mobile examination center. DXA accurately detects fat and muscle, providing information on total and regional percentages of fat and muscle composition. The KNHANES datasets included whole-body DXA measurements of bone mineral density, diagnosis of osteoporosis, and fat percentage (fat mass/total mass × 100). In this study, the diagnosis of osteoporosis and fat percentage were used in the analysis.

In addition, this study evaluated social characteristics based on questionnaires across three domains: smoking, alcohol intake, and physical activity, all of which directly influence an individual’s health status^11^. Smoking was quantified as cumulative exposure over an individual's lifetime, measured in pack-years. Pack-years, which has been used previously as a representative of smoking amount, were calculated by multiplying the number of packs of cigarettes smoked per day by the total number of years a person has been smoking^12^. Similarly, alcohol intake was determined by the weekly average amount of alcohol, measured in Soju bottles. Soju, the alcoholic beverage in Korea, has an alcohol content of approximately 16% and is packaged in 360 ml bottles^13^. Physical activity was assessed based on parameters from KNHANES questionnaires, converting them into metabolic equivalents (METs).

The assessment of hip radiographs utilized the Kellgren–Lawrence grading system. Two independent radiologists conducted evaluations for hip x-ray, and in cases where a one-grade discrepancy existed, the higher grade was deemed valid. Discrepancies surpassing one grade underwent a thorough review by a third radiologist, and the grade aligned with the third assessment was adopted. As in previous studies, Kellgren–Lawrence grades 2 to 4 were categorized as abnormal hip x-ray, while grades 0 to 1 were considered normal on hip x-ray^14,15^. The presence of hip joint-related pain was determined through a survey that asked about the experience of hip pain.

### Statistical analysis

#### Descriptive analysis

The baseline features of participants were assessed respectively for the male and female groups to detect differences in characteristics between the gender. A comparable analysis was conducted by dividing them into age groups: 50–59, 60–69, and 70–79. Variables such as weight, expressed as mean and standard deviation, underwent a Student’s *t*-test for comparison. Categorical variables, including radiographic hip x-ray grade, were presented as percentages and numerical values, and the chi-squared test was conducted for comparisons. All variables underwent tests for normality, and in cases where normality was not observed, non-parametric tests, such as Mann–Whitney U test, were conducted for each respective variable.

#### Propensity score matching

Previous studies and the descriptive analysis of this study suggested that sex and age are important factors in hip pain. We employed the propensity score (PS) matching method to reduce confounding bias related to sex and age^16,17^. A 1:1 ratio between groups was established to enhance similarity between male and female groups. Similar to previous studies, the standardized mean difference (SMD) was used to assess the quality of pairing, with an SMD less than 0.1 considered well-paired^18^. After matching for sex and age, participant characteristics were compared using the Student’s t-test for continuous variables and the chi-squared test for categorical variables. In cases where normality was not observed, non-parametric tests, such as Mann–Whitney U test, were conducted for each respective variable.

We conducted all analyses using R version 4.2.2 on the Windows 10 operating system, utilizing several R packages such as ‘dplyr,’ ‘moonBook,’ and related libraries. Statistical significance was defined as p < 0.05 for all tests conducted.

### Ethics approval

This study was based on the KNHANES database, which was approved by the Institutional Review Board (IRB) of the KDCA. We obtained exemption for informed consent from the Public Institutional Review Board Designated by the Ministry of Health and Welfare (IRB No. 2024-0180-001, Approval No. P01-202402-01-011).

All analyses were performed in accordance with relevant guidelines and regulations, and informed consent was obtained from all participants.

## Result

### Correlation among variables

Supplementary Table S1 presents the correlation matrix of all characteristics utilized in this study. A correlation coefficient closer to 1 indicates a strong positive linear relationship, while closer to -1 indicates a strong negative linear relationship. A correlation coefficient near zero suggests no discernible relationship between the variables. Hip pain and abnormal hip x-ray show a significant positive correlation (coefficient = 0.06, p < 0.001). Additionally, aging and female sex were associated with hip pain (coefficients = 0.141 and 0.139, respectively, p < 0.001).

### Descriptive analysis

Descriptive analysis provides an overview of characteristic features and differences between sexes (Table 1). Considering the entire Korean population, 864,792 (9.7%) of Koreans are experiencing hip pain, while 87,098 (1.0%) show abnormal hip x-ray. Female participants were diagnosed more frequently with hypertension, dyslipidemia, cancer, osteoporosis, and depression. However, diabetes and heart disease were more frequent in male participants. Female participants tended to be older, shorter, lighter, and had a higher percentage of fat composition. Less engagement in blue-collar occupations and lower income were associated with female participants. Social factors such as alcohol intake, smoking, and physical activity were lower in female participants. The feeling of hip pain was higher in female participants, although abnormal hip x-ray was more frequent in male participants, implying a mismatch between pain and plain radiography.

**Table 1:** Descriptive analysis of participants by sex.

	Total (N = 8,898,044)	Male (N = 4,203,881)	Female (N = 4,694,163)
Age	61.0 ± 8.2	60.4 ± 7.9*	61.4 ± 8.5*
Height (cm)	160.4 ± 8.8	167.4 ± 5.9*	154.1 ± 5.7*
Weight (kg)	62.1 ± 10.0	66.8 ± 9.5*	57.8 ± 8.5*
BMI	24.1 ± 3.1	23.8 ± 2.9*	24.3 ± 3.2*
Recent weight gain	10.6%	7.6%*	13.4%*
Occupation type (blue collar)	46.1%	57.5%*	35.9%*
Household income (top 50%)	45.4%	50.7%*	40.6%*
Diagnosis of hypertension	37.4%	33.6%*	40.7%*
Diagnosis of dyslipidemia	16.8%	13.4%*	19.9%*
Diagnosis of diabetes	13.9%	15.4%*	12.5%*
Diagnosis of kidney disease	0.5%	0.3%*	0.7%*
Diagnosis of heart disease	2.9%	4.7%*	4.5%*
Diagnosis of cancer	4.9%	3.9%*	5.8%*
Menopause	48.4%	0*	91.7%*
Depressive mood (> 14 days)	15.5%	10.8%*	19.8%*
Alcohol intake	0.8 ± 1.6	1.6 ± 2.1*	0.2 ± 0.6*
Smoking amount	11.8 ± 18.9	23.9 ± 21.1*	1.1 ± 5.7*
Physical activity	271.0 ± 489.9	303.9 ± 507.6*	58.8 ± 12.4*
Fat composition (%)	29.2 ± 8.1	22.7 ± 5.1*	35.0 ± 5.4*
Diagnosis of osteoporosis	9.5%	1.5%*	16.6%*
Hip pain	9.7%	5.4%*	13.6%*
Abnormal hip x-ray	1.0%	1.4%*	0.6%*

Household income, top 50% household income; Heart disease, cardiovascular disease; Depressive Mood, feeling depressed for more than 2 weeks; Alcohol intake, number of soju bottle consumed per week; Smoking amount, average number of cigarette packs smoked per day was based on the number of years of smoking; METs, based on questionnaires; Abnormal hip x-ray, radiologically more than moderate based on the Kellgren–Lawrence grading system.

*BMI* body mass index.

*Indicate p < 0.001.

This study also assessed the age distribution of the participants, analyzing three groups: ages 50–59, 60–69, and 70–79 (Table 2). As expected, older individuals exhibited a higher prevalence of medical complications, including menopause, hypertension, diabetes, heart disease, cancer, and osteoporosis. The aging process was associated with various factors such as shorter height, lesser weight, and increased fat composition. Less engagement in blue-collar occupations and lower income were associated with aging. In social aspects, physical activity and alcohol intake decreased, while smoking amount increased with aging. Furthermore, the sensation of hip pain was correlated with aging, although no significant association was observed between aging and abnormal hip x-ray.

**Table 2:** Descriptive analysis of participants by age.

	Age group 50–59 (N = 4,548,470)	Age group 60–69 (N = 2,630,595)	Age group 70–79 (N = 1,718,979)
Age	54.1 ± 2.8*	64.4 ± 2.9*	73.9 ± 2.7*
Sex (female)	50.6%*	52.1%*	59.5%*
Height (cm)	162.2 ± 8.5*	160.0 ± 8.3*	156.2 ± 8.8*
Weight (kg)	63.6 ± 9.9*	62.1 ± 9.8*	58.1 ± 9.7*
BMI	24.1 ± 2.8	24.2 ± 3.2	23.8 ± 3.3
Recent weight gain	14.2%*	7.7%*	5.7%*
Occupation type (Blue collar)	51.5%*	47.2%*	30.2%*
Household income (top 50%)	61.1%*	35.0%*	19.7%*
Diagnosis of hypertension	26.9%*	44.6%*	54.2%*
Diagnosis of dyslipidemia	15.1%*	21.1%*	14.9%*
Diagnosis of diabetes	10.2%*	15.9%*	20.4%*
Diagnosis of kidney disease	0.4%*	0.7%*	0.3%*
Diagnosis of heart disease	3.0%*	6.0%*	6.7%*
Diagnosis of cancer	3.9%*	5.6%*	6.5%*
Menopause	42.0%*	52.1%*	59.5%*
Depressive mood (> 14 days)	16.5%*	14.3%*	15.0%*
Alcohol intake	1.1 ± 1.8*	0.7 ± 1.5*	0.5 ± 1.3*
Smoking amount	11.4 ± 16.7*	12.0 ± 20.6*	12.7 ± 21.4*
Physical activity	316.1 ± 513.3*	250.0 ± 461.8*	183.9 ± 453.0*
Fat composition (%)	28.7 ± 7.9*	29.5 ± 8.2*	30.2 ± 8.2*
Diagnosis of osteoporosis	4.9%*	12.5%*	17.1%*
Hip pain	6.7%*	10.4%*	16.7%*
Abnormal hip x-ray	1.0%*	0.5%*	1.6%*

Household income, top 50% household income; Heart disease, cardiovascular disease; Depressive Mood, feeling depressed for more than 2 weeks; Alcohol intake, number of soju bottles consumed per week; Smoking amount, average number of cigarette packs smoked per day was based on the number of years of smoking; METs, based on questionnaires; Abnormal hip x-ray, radiologically more than moderate based on the Kellgren–Lawrence grading system.

*BMI* body mass index.

*Indicate p < 0.001.

### Epidemiology of hip pain: controlling for confounding bias of age and sex

Our results suggested that age and sex influenced multiple characteristics, including hip pain, radiologic hip X-ray, and various epidemiologic factors. After PS matching for age and sex, we identified 864,792 participants who experienced hip pain and another 864,792 participants who did not feel hip pain (Table 3). Both groups exhibited a similar distribution of age (64.5 ± 8.8 years) and sex (73.8% of female ratio). The diagnosis of diabetes (15.3 vs 17.5%) and heart disease (5.9 vs 6.7%) was associated with experiencing hip pain (p < 0.001). Depressive mood was linked to hip pain (13.6 vs 24.5%). Osteoporosis (14.5 vs 24.1%) and higher weight (59.5 vs 59.7 kg) were more likely to be associated with hip pain. Working in a blue-collar occupation (25.6 vs 37.9%) and having lower household income (42.6 vs 32.3%) were also linked to hip pain (p < 0.001). Regarding social factors, there was a correlation between high alcohol intake, smoking amount, and physical activities with hip pain (p < 0.001).

**Table 3:** PS matching for age and sex to find out characteristic difference of feeling hip pain.

	No hip pain (N = 864,792)	Feeling hip pain (N = 864,792)
Age	64.5 ± 8.8	64.5 ± 8.8
Sex (female)	73.8%	73.8%
The standardized mean difference (SMD) of age and sex < 0.001		
Height (cm)	157.2 ± 8.2*	156.6 ± 8.7*
Weight (kg)	59.5 ± 9.0*	59.7 ± 10.5*
BMI	24.0 ± 2.9*	24.3 ± 3.6*
Recent weight gain	11.6%*	11.3%*
Occupation type (blue collar)	25.6%*	37.9%*
Household income (top 50%)	42.6%*	32.3%*
Diagnosis of hypertension	45.8%*	43.0%*
Diagnosis of dyslipidemia	21.5%*	19.8%*
Diagnosis of diabetes	15.3%*	17.5%*
Diagnosis of kidney disease	0.5%*	1.3%*
Diagnosis of heart disease	5.9%*	6.7%*
Diagnosis of cancer	9.3%*	6.8%*
Menopause	69.8%	69.9%
Depressive mood (> 14 days)	13.6%*	24.5%*
Alcohol intake	0.4 ± 1.2*	0.6 ± 1.4*
Smoking amount	7.2 ± 16.6*	7.6 ± 16.1*
Physical activity	209.5 ± 440.4*	277.2 ± 545.8*
Fat composition (%)	32.3 ± 7.0*	31.9 ± 8.3*
Diagnosis of osteoporosis	14.5%*	24.1%*
Abnormal hip x-ray	0.6%*	2.8%*

Household income, top 50% household income; Heart disease, cardiovascular disease; Depressive Mood, feeling depressed for more than 2 weeks; Alcohol intake, number of soju bottles consumed per week; Smoking amount, average number of cigarette packs smoked per day was based on the number of years of smoking; METs, based on questionnaires; Abnormal hip x-ray, radiologically more than moderate based on the Kellgren–Lawrence grading system.

*BMI* body mass index.

*Indicate p < 0.001.

### Epidemiology of abnormal hip x-ray: controlling for confounding *bias* of age and sex

To find out characteristic difference of advanced hip x-ray, we reduced confounding bias made by age and sex. After PS matching, we found 87,098 participants with advanced hip x-ray and another 87,098 participants with normal hip x-ray (Table 4). Two groups showed same distribution of age (62.4 ± 9.5 years) and sexes (30.3% of female ratio). The diagnosis of diabetes (8.9 vs 25.0%) and depressive mood (10.2 vs 29.6%) were associated with advanced hip x-ray (p < 0.001). Excessive weight (63.0 vs 66.8 kg), BMI (23.4 vs 24.4), and higher fat composition (26.2 vs 28.2%) showed a correlation with abnormal hip x-rays (p < 0.001). In terms of social factors, increased smoking was found to be linked with abnormal hip x-rays, while lower physical activity was associated with abnormal hip X-rays (p < 0.001).

**Table 4:** PS matching for age and sex to find out characteristic difference of abnormal hip X-ray.

	Normal hip X-ray (N = 87,098)	Abnormal hip X-ray (N = 87,098)
Age	62.4 ± 9.5	62.4 ± 9.5
Sex (female)	30.3%	30.3%
The standardized mean difference (SMD) of age and sex < 0.001		
Height (cm)	163.7 ± 8.4*	165.4 ± 10.4*
Weight (kg)	63.0 ± 10.2*	66.8 ± 10.9*
BMI	23.4 ± 2.7*	24.4 ± 3.3*
Recent weight gain	8.1%*	9.2%*
Occupation type (blue collar)	27.4%*	46.0%*
Household income (top 50%)	49.3%*	42.1%*
Diagnosis of hypertension	47.7%*	43.6%*
Diagnosis of dyslipidemia	15.4%*	13.2%*
Diagnosis of diabetes	8.9%*	25.0%*
Diagnosis of Kidney disease	1.2%*	0%*
Diagnosis of heart disease	10.2%*	2.8%*
Diagnosis of Cancer	8.2%*	3.2%*
Menopause	27.4%*	25.0%*
Depressive mood (> 14 days)	10.2%*	29.6%*
Alcohol intake	1.3 ± 2.0	1.3 ± 2.0
Smoking amount	21.4 ± 25.2*	23.0 ± 28.9*
Physical activity	390.9 ± 799.0*	158.2 ± 223.7*
Fat composition (%)	26.2 ± 7.6*	28.2 ± 8.8*
Diagnosis of osteoporosis	4.6%*	11.2%*
Hip pain	9.2%*	27.6%*

Household income, top 50% household income; Heart disease, cardiovascular disease; Depressive Mood, feeling depressed for more than 2 weeks; Alcohol intake, number of soju bottles consumed per week; Smoking amount, average number of cigarette packs smoked per day was based on the number of years of smoking; METs, based on questionnaires; Abnormal hip x-ray, radiologically more than moderate based on the Kellgren–Lawrence grading system.

*BMI* body mass index.

*Indicate p < 0.001.

## Discussion

This study analyzed hip pain with a study group which has the representativeness of the entire South Korean population (N = 46,286,503). This study found that 864,792 Koreans (9.7% of the population) are currently experiencing hip pain, and 87,098 Koreans (1.0% of the population) show a high Kellgren-Lawrence grade of hip joint.

The decision to select participants aged 50 and above was made because KNHANES offers plain radiography examinations of hip joint only for individuals aged 50 and older. Additionally, we selected the age of study population under 80 because in this survey, individuals aged 80 and above were grouped into a single category, making precise evaluation difficult. While analyzing all age groups might have provided more meaningful results, the current study's age restrictions were necessary based on the available data.

Descriptive analysis revealed the distinct characteristics between sexes. Previous studies have emphasized the importance of understanding the sex-based differences in hip pain related diseases pathophysiology. In particular, female participants tend to experience a significant increase in the occurrence of knee, hip, and hand OA around menopausal years^19^. Previous research suggests that estrogens play a protective role against the damaging effects of reactive oxygen species and cytokines within the joint space, pointing to a multifaceted influence of hormones not just on disease emergence but on its progression as well^20,21^. Avascular necrosis, a significant contributor to hip pain, further exemplifies the complex interplay between hormonal changes and musculoskeletal diseases, with evidence suggesting its linkage to estrogen and progesterone levels affected by fat metabolism^22^. In the context of our study, which focuses on individuals aged 50 and above, the predominance of postmenopausal women (around 90%) is especially indicative. The hormonal changes associated with menopause, characterized by a significant drop in estrogen and related hormones, appear to predispose women to higher BMI, increased fat composition, and a greater risk of osteoporosis^23,24^. This pattern highlights not only the direct impact of hormonal changes on musculoskeletal health but also the broader implications of these changes on women's overall health post-menopause. It reinforces the necessity of adopting a gender-specific approach in both research and treatment strategies for musculoskeletal diseases, considering the profound effects of hormonal changes experienced by women during and after the menopausal transition.

Additionally, descriptive analysis of this study found a correlation between the hip pain and aging, although no distinct association was observed between aging and the presence of abnormaliy on hip plain radiography. Previous studies mentioned the discrepancy of Kellgren-Lawrence grade ≥ 2 and hip pain^25,26^. Hip pain arises from various causes, including intra-articular factors like labral tears and chondromalacia, extra-articular factors such as tendinopathy and bursitis, and referred pain originating from sources like the sacroiliac joint and spine^27^. Hip plain radiography enables the visualization of changes in bone structure, joint space, and surrounding tissues. However, observing soft tissues, including tendons, nerves, and muscles through hip X-rays is challenging^28^. While conditions like labral tear and tendinopathy may be indirectly reflected through joint space narrowing or alterations in bone margins on x-rays, the clinical information obtained from plain radiography is limited^29^. In actual clinical practice, magnetic resonance imaging (MRI) is utilized to supplement radiographic findings, providing a detailed examination of soft tissues^30^.

This study included multiple factors for hip pain, and suggested that heart disease and diabetes were significantly associated with hip pain. Previous studies from United States showed that diagnosis of OA was associated with diagnosis of heart failure, and another studies from China assessed that diagnosis of spine OA was associated with myocardial infarction^31,32^. In addition, diabetes was known for causing oxidative stress and low-grade chronic inflammation, which led to OA and avascular necrosis of femoral head^33^. To explain association between heart disease, diabetes, and hip pain, insufficient blood supply can be an important factor. Vasoconstriction due to diabetes and decreased cardiac output by heart disease can cause insufficient blood supply to lower limbs^34,35^. Less blood supply to hip joint space causes chronic change, such as decreased synovial fluid, joint space narrowing, and subchondral change, which make hip pain more severe^34^.

Lower household income and engagement in blue-collar occupations were found to be associated with hip pain and abnormal hip x-ray. The connection between low socio-economic status and a heightened prevalence of OA has been extensively examined in previous research. A cross-sectional study conducted in the United States revealed that a lower educational status, non-managerial occupational status, and a higher poverty rate were correlated with an elevated prevalence of osteoarthritis^36^. Consistent with prior studies in the United States, limited access to medical facilities and insufficient coverage from medical insurance were identified as contributors to the increased prevalence of joint pain^37^. Nevertheless, it is worth noting that the national health insurance system covers approximately 97% of the population, and the remaining 3% are supported by a tax-funded program to ensure access for low-income citizens in Korea^38^. Consequently, poor access to medical facilities or inadequate insurance does not accurately reflect the situation in Korea. Instead, it is worth considering alternative factors, such as individuals engaged in blue-collar occupations who may not fully recognize the necessity of medical services, leading to a reluctance to seek medical attention^39^. Further research will be pursued to substantiate the relationship between low socioeconomic status and the heightened prevalence of hip pain and advanced hip plain radiography findings.

Multiple nationwide studies have been conducted to explore various aspects of hip pain. For instance, studies involving Australians evaluated age, sex, residency, and treatment options, while studies of Americans aimed to explore associations with sarcopenia, age, sex, race, and education level^40,41^. However, most of these studies were based on regional cohorts, and few employed sampling weights to accurately represent an entire national population. Furthermore, past research primarily focused on a few epidemiologic factors, such as age, sex, and occupation, and lacked data on characteristics of hip pain, such as subjective pain perception and objective radiographic grade. This study utilized sampling weights to reflect the entire population of Korea and analyzed various aspects of Koreans, including medical, demographic, mental, social, and musculoskeletal factors, to identify characteristics of hip pain in Korea.

Diabetes, a metabolic disorder, elevates levels of free plasma fatty acids and creates a pro-inflammatory state, leading to alterations in blood vessels^42^. This process can reduce blood flow to the femoral head, increasing vulnerability, especially in patients with inflammatory diseases like osteoarthritis, and contributing to hip pain and abnormal x-ray results. Additionally, obesity, quantified as BMI in this study, further exacerbates the inflammatory state. Our study supports these findings^43^. Alcohol consumption has also been linked to hip fracture in several previous studies, showing that alcohol-induced production of circulating pro-inflammatory cytokines and C-reactive protein elevates inflammation levels and worsens hip pain^44^.

The apparent association between hip pain sensation and alcohol consumption or smoking status was assessed. Previous studies have demonstrated that smoking and alcohol intake contribute to cartilage loss, immune system modulation, chronic inflammation, and exacerbation of hip pain^45,46^. Our findings reveal that the subjective experience of hip pain is correlated with both alcohol consumption and smoking status, suggesting the importance of modifying smoking and drinking habits for hip pain prevention. Regarding physical activities, this study showed that participants with higher levels of activity are associated with experiencing hip pain. The results seem reasonable, as increased physical activity is associated with a higher burden on the hip joint^17^.

Participants with abnormal hip x-ray and those with normal hip x-ray exhibited statistically insignificant differences in alcohol intake. However, a higher level of smoking was correlated with abnormal hip X-ray. Previous studies have suggested that heavy smokers had a lower risk of osteoarthritic change at four sites (knee, hand, foot, and spine), which could be explained by nicotine’s upregulation of glycosaminoglycan and collagen synthetic activity in joint chondrocytes^47,48^. This study suggested that unlike other joint, severe hip pain is associated with heavy smoking. Regarding physical activities, the expectation was that increasing physical activity would lead to more hip joint damage, resulting in the manifestation of advanced hip x-rays. However, this study revealed that lower levels of physical activity were associated with abnormal hip X-ray. This result suggests that participants with abnormal hip x-ray frequently reported experiencing hip pain, leading to restricted physical activity. The challenge in interpreting causation arises due to the cross-sectional study design.

Our study has certain limitations. First of all, the KNHANES recruits 10,000 participants annually, covering 3,840 households in 192 regions of South Korea. While this sampling method is known to statistically represents entire Korean population, the relatively low prevalence of hip pain in Korea led to inaccuracies in our study. Specifically, our study initially enrolled 3,896 participants before weight calculation, which was then extrapolated to 8,898,044 participants after applying sampling weights. This implies that each individual's result can have a significant impact, even though its accuracy and reasonability of calculation. Additionally, as mentioned earlier, KNHANES utilized a cross-sectional design, establishing associations rather than causation. Furthermore, if the survey had included questions on the intake of nutrients involved in bone formation, such as Vitamin D, or trauma history like slips and falls, more in-depth research on hip pain and x-ray results would have been possible. This limitation arose because KNHANES did not include such questions. Moreover, as pointed out, smoking and physical activities are based on self-reported data from questionnaires, which can introduce various biases, including recall bias. In addition, determining the presence of hip osteoarthritis as past medical history could provide additional objective information regarding hip pain and x-ray results. However, including this aspect was challenging as KNHANES does not include such questions. We anticipate that future prospective studies using nutritional characteristics, trauma events, and other objective numerical variables such as wearable devices data to monitor physical activities, will further support our research and lead to a more comprehensive understanding of hip pain. Finally, the last limitation of our study to be mentioned is the reliability and repeatability of the Kellgren-Lawrence grade for hip X-rays. While inherent in a grading system based on X-rays, even with grading conducted by two or three radiologists as in the KNHANES design, errors in this classification method can still occur. In future research, adopting AI-based grading system with validated repeatability and reliability could result in more solid assessment approach.

## Conclusions

This study explores hip pain across the entire Korean population (N = 46,286,503) using the KNHANES V-5 dataset. We found that 9.7% of Koreans (864,792 individuals) experience hip pain, and 1.0% (87,098 individuals) exhibit a high Kellgren-Lawrence grade of hip plain radiography. Sex disparities indicate that abnormal hip x-ray is more prevalent in males, while females report higher hip pain. Aging is associated with more hip pain, an increased prevalence of various medical complications, and a reduced lifestyle. Propensity score matching for age and sex identifies positive associations between hip pain and characteristics such as weight, BMI, diabetes, heart disease, depressive mood, and osteoporosis. Hip pain correlates with high alcohol intake, smoking, and physical activity. Similarly, abnormal hip x-rays are linked to excessive weight, BMI, higher fat composition, increased smoking, and lower physical activity. Alcohol intake exhibits no significant association with abnormal hip x-rays. The study highlights the need for a personalized approach to managing hip pain, considering demographic, medical, social, and even mental factors in the Korean population.

### Supplementary Information

Supplementary Table S1.

## Data Availability

All KNHANES dataset files are available on the following KNHANES website: https://knhanes.kdca.go.kr/knhanes/sub03/sub03_02_05.do.
