# Supplementary material for: Insights into Hip pain using Hip X-ray: Epidemiological study of 8,898,044 Koreans
Source: Sci Rep. 2024 Aug 21;14:19405. doi: 10.1038/s41598-024-70259-z (PMC11339292; doi:10.1038/s41598-024-70259-z)
Supplement: Supplementary file 1 — Supplementary Table S1. [file 41598_2024_70259_MOESM1_ESM.docx]

**Supplementary Table S1**. Correlation matrix of all variables used in this study

|  | **Age** | **Sex** | **Height** | **Weight** | **BMI** | **weight gain** | **Blue collar** | **Household income** | **Hypertension** | **Dyslipidemia** | **Diabetes** | **Kidney disease** | **Heart disease** | **Cancer** | **Menopause** | **Depressive Mood** | **Alcohol intake** | **Smoking amount** | **Physical activity** | **Fat composition** | **Osteoporosis** | **Hip pain** | **hip X-ray** |
| --- | --- | --- | --- | --- | --- | --- | --- | --- | --- | --- | --- | --- | --- | --- | --- | --- | --- | --- | --- | --- | --- | --- | --- |
| **Age** | 1.000 | 0.060* | -0.271* | -0.213* | -0.036* | -0.123* | -0.147* | -0.351* | 0.241* | 0.027* | 0.130* | 0.004* | 0.085* | 0.052* | 0.160* | -0.028* | -0.137* | 0.019* | -0.111* | 0.068* | 0.178* | 0.141* | 0.017* |
| **Sex** | 0.060* | 1.000 | -0.755* | -0.448* | 0.087* | 0.093* | -0.216* | -0.102* | 0.073* | 0.086* | -0.041* | 0.029* | -0.007* | 0.043* | 0.916* | 0.123* | -0.436* | -0.603* | -0.064* | 0.761* | 0.256* | 0.139* | -0.045* |
| **Height** | -0.271* | -0.755* | 1.000 | 0.625* | -0.071* | -0.031* | 0.142* | 0.205* | -0.104* | -0.053* | -0.007* | -0.029* | 0.005* | -0.032* | -0.717* | -0.087* | 0.334* | 0.460* | 0.077* | -0.583* | -0.238* | -0.140* | 0.056* |
| **Weight** | -0.213* | -0.448* | 0.625* | 1.000 | 0.729* | 0.106* | 0.061* | 0.195* | 0.121* | 0.084* | 0.063* | -0.004* | 0.038* | -0.061* | -0.435* | -0.074* | 0.215* | 0.250* | 0.073* | -0.033* | -0.173* | -0.079* | 0.047* |
| **BMI** | -0.036* | 0.087* | -0.071* | 0.729* | 1.000 | 0.165* | -0.049* | 0.063* | 0.244* | 0.152* | 0.084* | 0.025* | 0.046* | -0.049* | 0.069* | -0.017* | -0.020* | -0.078* | 0.018* | 0.472* | -0.016* | 0.020* | 0.010* |
| **weight gain** | -0.123* | 0.093* | -0.031* | 0.106* | 0.165* | 1.000 | -0.052* | 0.045* | -0.006* | 0.047* | -0.059* | 0.024* | -0.038* | -0.004* | 0.068* | 0.044* | -0.051* | -0.047* | 0.017* | 0.153* | -0.017* | 0.007* | -0.005* |
| **Blue collar** | -0.147* | -0.216* | 0.142* | 0.061* | -0.049* | -0.052* | 1.000 | 0.032* | -0.105* | -0.127* | -0.056* | -0.048* | -0.063* | -0.077* | -0.222* | -0.054* | 0.133* | 0.103* | 0.111* | -0.252* | -0.056* | -0.054* | 0.000 |
| **Household income** | -0.351* | -0.102* | 0.205* | 0.195* | 0.063* | 0.045* | 0.032* | 1.000 | -0.079* | 0.014* | -0.056* | -0.015* | -0.029* | 0.016* | -0.131* | -0.073* | 0.057* | 0.002* | 0.062* | -0.039* | -0.068* | -0.086* | -0.006* |
| **Hypertension** | 0.241* | 0.073* | -0.104* | 0.121* | 0.244* | -0.006* | -0.105* | -0.079* | 1.000 | 0.233* | 0.189* | 0.028* | 0.106* | 0.007* | 0.108* | 0.034* | -0.036* | -0.030* | -0.053* | 0.192* | 0.055* | 0.038* | 0.013* |
| **Dyslipidemia** | 0.027* | 0.086* | -0.053* | 0.084* | 0.152* | 0.047* | -0.127* | 0.014* | 0.233* | 1.000 | 0.198* | 0.041* | 0.106* | 0.005* | 0.099* | 0.073* | -0.058* | -0.046* | -0.030* | 0.132* | 0.067* | 0.026* | -0.010* |
| **Diabetes** | 0.130* | -0.041* | -0.007* | 0.063* | 0.084* | -0.059* | -0.056* | -0.056* | 0.189* | 0.198* | 1.000 | 0.025* | 0.051* | -0.006* | -0.014* | 0.043* | 0.001* | 0.085* | -0.030* | -0.008* | 0.023* | 0.034* | 0.032* |
| **Kidney disease** | 0.004* | 0.029* | -0.029* | -0.004* | 0.025* | 0.024* | -0.048* | -0.015* | 0.028* | 0.041* | 0.025* | 1.000 | 0.000 | -0.016* | 0.035* | 0.056* | -0.026* | -0.006* | -0.025* | 0.042* | 0.042* | 0.036* | -0.007* |
| **Heart disease** | 0.085* | -0.007* | 0.005* | 0.038* | 0.046* | -0.038* | -0.063* | -0.029* | 0.106* | 0.106* | 0.051* | 0.000 | 1.000 | 0.019* | 0.007* | 0.021* | -0.013* | 0.080* | 0.010* | 0.026* | 0.028* | 0.034* | -0.009* |
| **Cancer** | 0.052* | 0.043* | -0.032* | -0.061* | -0.049* | -0.004* | -0.077* | 0.016* | 0.007* | 0.005* | -0.006* | -0.016* | 0.019* | 1.000 | 0.061* | 0.041* | -0.073* | -0.023* | -0.035* | 0.019* | 0.046* | 0.029* | -0.008* |
| **Menopause** | 0.160* | 0.916* | -0.717* | -0.435* | 0.069* | 0.068* | -0.222* | -0.131* | 0.108* | 0.099* | -0.014* | 0.035* | 0.007* | 0.061* | 1.000 | 0.115* | -0.402* | -0.549* | -0.072* | 0.700* | 0.273* | 0.141* | -0.046* |
| **Depressive Mood** | -0.028* | 0.123* | -0.087* | -0.074* | -0.017* | 0.044* | -0.054* | -0.073* | 0.034* | 0.073* | 0.043* | 0.056* | 0.021* | 0.041* | 0.115* | 1.000 | -0.020* | -0.042* | 0.011* | 0.068* | 0.060* | 0.081* | 0.039* |
| **Alcohol intake** | -0.137* | -0.436* | 0.334* | 0.215* | -0.020* | -0.051* | 0.133* | 0.057* | -0.036* | -0.058* | 0.001* | -0.026* | -0.013* | -0.073* | -0.402* | -0.020* | 1.000 | 0.332* | 0.043* | -0.341* | -0.125* | -0.058* | 0.026* |
| **Smoking**  **amount** | 0.019* | -0.603* | 0.460* | 0.250* | -0.078* | -0.047* | 0.103* | 0.002* | -0.030* | -0.046* | 0.085* | -0.006* | 0.080* | -0.023* | -0.549* | -0.042* | 0.332* | 1.000 | 0.056* | -0.463* | -0.115* | -0075* | 0.059* |
| **Physical activity** | -0.111* | -0.064* | 0.077* | 0.073* | 0.018* | 0.017* | 0.111* | 0.062* | -0.053* | -0.030* | -0.030* | -0.025* | 0.010* | -0.035* | -0.072* | 0.011* | 0.043* | 0.056* | 1.000 | -0.060* | -0.035* | 0.004* | -0.023* |
| **Fat composition** | 0.068* | 0.761* | -0.583* | -0.033* | 0.472* | 0.153* | -0.252* | -0.039* | 0.192* | 0.132* | -0.008* | 0.042* | 0.026* | 0.019* | 0.700* | 0.068* | -0.341* | -0.463* | -0.060* | 1.000 | 0.196* | 0.107* | -0.012* |
| **Osteoporosis** | 0.178* | 0.256* | -0.238* | -0.173* | -0.016* | -0.017* | -0.056* | -0.068* | 0.055* | 0.067* | 0.023* | 0.042* | 0.028* | 0.046* | 0.273* | 0.060* | -0.125* | -0.155* | -0.035* | 0.196* | 1.000 | 0.164* | 0.006* |
| **Hip pain** | 0.141* | 0.139* | -0.140* | -0.079* | 0.020* | 0.007* | -0.054* | -0.086* | 0.038* | 0.026* | 0.034* | 0.036* | 0.034* | 0.029* | 0.141* | 0.081* | -0.058* | -0.075* | 0.004* | 0.107* | 0.164* | 1.000 | 0.060* |
| **Abnormal hip X-ray** | 0.017* | -0.045* | 0.056* | 0.047* | 0.010* | -0.005* | 0.000 | -0.006* | 0.013* | -0.010* | 0.032* | -0.007* | -0.009* | -0.008* | -0.046* | 0.039* | 0.026* | 0.059* | -0.023* | -0.012* | 0.006* | 0.060* | 1.000 |
